# Topics of Public Interest

**Published:** 1905-11

**Authors:** 


### TOPICS OF PUBLIC INTEREST.

### The Sorrows of Sato.

### Japanese and American Journalists.

By A. IMARA SATO, First Secretary of the Japanese Peace Commission.

[*Sunday Magazine of the New York Tribune.*]

THE Japanese journalist will never, I fear, reach the proud preëminence of his American confrère. He will never be so superb, so royal, so all-commanding. It is a pity, but it is so. The American journalist meets the arriving stranger with a condescending hospitality and proprietary patronage that is impressive, and should he approve of the stranger, makes him at home in a country which the journalist appears to own. With us this cannot be. Our journalist is not yet monarch of all he surveys. The Emperor and the elder statesmen still have something to say.

It is an interesting question, this freedom of the press under the Republic. It is particularly interesting to us of Japan, because it is a social problem which we must adjust. Journalism with us is a new idea. It is more purely new, more entirely foreign perhaps, than any other idea which we have adopted from Western sources. Thirty years ago our people did not know such a thing as a newspaper, in the Occidental sense, existed. Today we have many newspapers of all classes, from the conservative to the "yellow." *The Jiji* of Tokio has, I think, a hundred thousand daily subscribers. Our people have developed a most eager taste for the daily newspaper. Consequently, the difference between our conditions and the American, between our journalists and yours, may be of interest. But as I said at first, in this parting message to my good friends the American press men, our journalists will never own our country in fee simple. We need it for other purposes.

Upon our arrival in this country we were courteously received by the advance guard of the press. It being my official duty to meet the journalists, to talk a great deal and say nothing in particular, I did so to the best of my ability. Therein the marvelous hypnotic power of the American journalist became quickly apparent. He makes people say what he wishes them to say. My unconscious tongue lent itself to so many statements of which I had no waking memory whatever that it was a continous surprise. When I inquired concerning this from one of the members of the press, he gravely assured me that a mail-order course in hypnotism was a requisite for every reporter before he became an interviewer. Is this not wonderful? And what hypnotisers they become in interviewing public men, and how rapidly practice perfects! Whether or not we shall adopt this hypnotic training in Japan is open to question. Our people are quick and sensitive and prone to act on impulse. So also are the authorities when journalists become too enterprising. American journalism will ever, I fear, be exotic in Japan.

But it is none the less exceedingly informing. One learns so much about himself. "Know thyself," said the Grecian sage; and after graduating from an American university and pondering this maxim all my life I had to come to America to learn many things about myself which I had never suspected. I was "the suave Sato," "the garrulous Sato." I did not converse; I "spoke my piece." I did not smoke a cigarette; I "lighted a torch" or "burnt a punk." I was not described; I was "Bertillonised" by the inquisitor of the fountain pen. I "pulled my fingers till the joints cracked, and replied with much dignity." With a high respect for the English language, I said "Come off!" "We are up against it;" and described an eminent Russian minister plenipotentiary as an "Also Ran." If we introduce hypnotism into Japanese journalism we shall compel it, I think, to temper truth with mercy.

But the American journalist has a range of fancy and a command of language which at times rises to the obscure. It is like certain eminent but involved English classics. One of them said:

"If Mr. Sato is to be considered the frivolous cut-up of the Japanese plenipotentiary outfit, the other roystering blades—his associates—must be veritable blue-points, and it is depressing to think of the mad frolic to which they will doubtless treat Messrs. Witte and Rosen at the dove-bake at Portsmouth."

Such sentences as this, and they occurred frequently, resisted the entire intellectual translating energy of our staff. The amicable relations now existing between Japan and the United States might not be sundered by a literal translation, but they might be strained. An "outfit," we discovered, was an assemblage of cow-boys with ropes and branding irons. Had such a thing as this been actually sent by the honorable people of Japan? It is true that we were uncommunicative; but then were we actually oysters? And "dove-bake"! What were we to make of that? The dove is found in our groves and in our poetry, but never in our ovens. Was the precious bird of peace to be regarded as cooked? Was the peace "cooked up" by us.

It gradually dawned upon us that instead of being a company of serious persons, engaged upon a mission of the highest diplomatic importance, we were a company of light comedians who had come abroad for the amusement of the American populace; that we were in a country of eighty million sovereigns and no king; and that the first duty of journalism was to make their millions of majesties laugh. They did it so well that they even made us laugh too. They were undeniably funny, the remarks and the cartoons. And they were undeniably impartial. If they reveled in the curve of our minister plenipotentiary's forehead, they took no less delight in the angle of the Russian plenipotentiary's nose. The primary duty of the journalist, it appears, was to amuse the people. There were eight Japanese journalists with us, representing eight of our leading papers. Our people love to laugh, and it may be recorded that our journalists made a careful note of this new and important principle.

We were regretfully convinced, however, that the Japanese journalist will always be retarded, if not greatly handicapped in his development, by courtesy. Courtesy is a part of his national tradition, and politeness is his national inheritance.

That this will ever be a clog upon the wheels of our advancing journalism became ever more certain. Only the American kings can rise superior to all forms. When a Japanese journalist interviews any person it is quite a formal proceeding. The conventions of social intercourse obtain here as elsewhere. If he has called upon a superior, official or social, the president of a bank for instance, he addresses him as "Anaki." If an equal, as an employee, the address is "Kimi"; if an inferior, as a laborer or peddler, it is "Omei." No such forms prevail in this country. If they did, the bank president would doubtless say "Anaki" to the journalist.

When a Japanese journalist comes to interview me he begins by a bow—"jack-knife," your journalists call it—as a salutation. I bow also. He says: "Good-morning, Mr. Sato, I hope that you are in good health." I reassure him on this point with equal formality. He says: "I trust I do not find you too much occupied for a few moments' important conversation?" I insist that my time is wholly his. He says: "Your friend Mr. So-and-so has very kindly given me a letter to you, and the object of my visit is—" etc., etc.

This, you see, is in strong contrast to the directness and simplicity of the American journalist. He may come in—providing you have met him before—and say, tilting a large cigar up at the conversational angle: "Hello, Sate! How's she heading? And how do you like swaying nations as a regular business?"

In thinking over this curious deficiency in our journalists I have come to the conclusion that it is essentially a question of money. Money is power, and the possession of it invariably gives a man a sense of power. We are informed that some of your journalists received as much as twenty-five thousand dollars a year. This surprised us a little, because up to that time we supposed that they all received that.

Our newspapers as a rule are poor. The war has been a heavy burden upon them, the cost of correspondents in the field and of long telegraph despatches being great. The reporter in Japan rarely receives more than twenty dollars a week. How can we hope that he will be arrogant and self-assured upon so small an income? Then too he is rarely a college graduate, with that limitless omniscience which developes, in the mind at least, after four years of a college course. He becomes a journalist after leaving the common school. Should journalism not satisfy his ambition, he abandons it for another vocation or becomes a writer of books. Our popular novels, however, sell for only twenty-five cents a copy, and it is long, as a rule, before the author buys a French automobile, a country-place and the controlling interest in a railroad. You see we have not railroads enough, as yet, to adequately equip our authors and our journalists according to rule. Journalists we have, men of prominence and influence, who possess the confidence of public men, but they have not yet undergone the true transformation. They are still as other men. They merely own their own papers and have not yet laid hands upon the country.

The exactions of Japanese journalism are, too, more exigent. The American journalist has merely to write his own language or that other language he has invented and uses in describing base-ball conflicts and physical battles in the arena. He is not required to furnish a key to his words, but the Japanese journalist must always write and print the key-word beside the other. Our papers are printed in Chinese characters. These are intelligible to the educated but not to the common people. Consequently you will always see in a popular Japanese paper beside each Chinese character—"the eloquent hen-track of Japan," as one of your journalists called it—a small character giving the sound. This is also a great handicap upon our journalism. How can we issue an "extra" containing a vivid account of something that has happened or that probably will happen, when we have no type alphabet but merely conventional ideographic blocks? We must arrange the event to suit the extra and cut the blocks beforehand; but to this eminence we have not, as yet, arisen. Still, we are a young country on modern lines and we may reasonably hope.

Our journalistic invention of word and phrase is as yet within strict limits. We cannot say "Casey flew to Riley; Mulligan walked; Murphy died; and McCarthy had Matty skinned to death." The key would be longer than the text, and besides it would first be necessary for us to understand it. The difficulties of high Occidental journalism are many. We do the best we can. "Telephone" we have made "den-wa" or "electric speech." "Phonograph" is "chi-ku-on-ki," or "store sound machine." "Submarine" we have made "sin-ko-sui-rai-tei" or "torpedo-boat which sinks in the sea." This method, however, is perhaps weak. It merely aims to be intelligible. With our evolution in base-ball and the sports will come that unintelligibility which is the American journalist's true power and his pride.

The American journalist is magnificently active. I cannot say of him at Portsmouth that: "He came, he heard, he conquered"; but I may say that he came, he did not hear, and he conquered all the same. The way in which the news leaked was astonishing. We would look askance at times at the eminent gentlemen from Russia, our eyes full of proper rebuke, and they would look at us at the same time with their eyes also full of equally proper rebuke. Nobody seemed to know exactly how the news got out. It seemed possible that to hypnotism the journalists added telepathy. The most startling occasion was when the local paper printed a despatch showing that the closely guarded treaty about to be signed had somehow reached New York and had been given out to the papers by a press association. This was staggering. We investigated, however, and found that Portsmouth was blameless. It had reached New York in a most roundabout way from a foreign land and was cabled back to the newspapers of that foreign land before they knew anything of its terms.

Activity our journalists possess; but our journalism needs many things. We need the patent pill to make the big advertisements. We are alas! a healthy nation and have not that rich superfluity in national diseases through which American journalists roll in gold. Our eminent physicians who cure people by the million at a dollar a bottle as yet practise only on the street corners and will not give to all the world their panaceas through the newspapers at hundreds of dollars a column. We have not yet the national pill, the national ointment or the national bakery, and our diagrams of impaired bodily functions are cofined to the medical schools. Still we have energy, ambition and the best of all models, and in time our journalist may reach the unique sovereignty of his American brother. So be it —and— "Sayonara."

